# Development of reference intervals for pupillometry in healthy dogs

**DOI:** 10.3389/fvets.2022.1020710

**Published:** 2022-10-26

**Authors:** Erinn P. Mills, Kelli Combs-Ramey, Grace P. S. Kwong, Daniel S. J. Pang

**Affiliations:** ^1^Western Veterinary Specialist and Emergency Centre, Calgary, AB, Canada; ^2^Calgary Animal Eye Care, Calgary, AB, Canada; ^3^Faculty of Veterinary Medicine, University of Calgary, Calgary, AB, Canada; ^4^Department of Community Health Sciences, Cumming School of Medicine, University of Calgary, Calgary, AB, Canada; ^5^Faculty of Veterinary Medicine, Université de Montréal, St Hyacinthe, QC, Canada

**Keywords:** pain, canine, pain evaluation, pupillometry, perioperative

## Abstract

**Background:**

Pupillometry, the measurement of pupil size and reactivity to a stimulus, has various uses in both human and veterinary medicine. These reflect autonomic tone, with the potential to assess nociception and emotion. Infrared pupillometry reduces inaccuracies that may occur when the pupillary light reflex is determined subjectively by the examiner. To our knowledge, there are no published studies outlining normal reference intervals for automated pupillometry in dogs.

**Objective:**

The objective of this study was to develop *de novo* automated pupillometry reference intervals from 126 healthy canine eyes.

**Methods:**

The pupillary light reflex (PLR) was measured with a handheld pupillometer (NeurOptics™ PLR-200™ Pupillometer). Parameters recorded included maximum pupil diameter (MAX), minimum pupil diameter (MIN), percent constriction (CON), latency (LAT), average constriction velocity (ACV), maximum constriction velocity (MCV), average dilation velocity (ADV) and time to 75% pupil diameter recovery (T75). One measurement was obtained for each eye.

**Results:**

The following reference intervals were developed: MAX (6.05–11.30 mm), MIN (3.76–9.44 mm), CON (−37.89 to −9.64 %), LAT (0.11–0.30 s), ACV (−6.39 to −2.63 mm/ s), MCV (−8.45 to −3.75 mm/s), ADV (−0.21–1.77 mm/s), and T75 (0.49–3.20 s).

**Clinical significance:**

The reference intervals developed in this study are an essential first step to facilitate future research exploring pupillometry as a pain assessment method in dogs.

## Introduction

Pupillometry is a measurement of the size and reactivity of the pupil to a stimulus, including assessment of the pupillary light reflex (PLR). Pupillometry is an important tool within both human and veterinary medicine as it is commonly used to assess ophthalmic function and neurological function in clinical patients. The PLR is the constriction of the pupil in response to light stimulating the retina. The PLR is mediated by a neural arc linking the optic nerve, optic chiasm, optic tract, pretectal area, and the parasympathetic nucleus of the oculomotor nerve. A synapse with the pupillomotor fibers of the oculomotor nerve elicits pupil contraction in response to light exposure *via* the smooth muscle of the iris sphincter (1). This reflex arc, being an integrated response of both the eye and the brain, allows assessment of iris innervation and function as well as ophthalmic and neurologic disease diagnosis in both human and veterinary medicine (2). Two opposing smooth muscles within the iris are responsible for constriction and dilation of the pupil, with the iris sphincter muscle being under parasympathetic control, and iris dilator muscle being under sympathetic control (3). Reflex pupil dilation can be evoked by acute pain and is a sympathetic response. Pain signals arising from noxious stimuli are conveyed to the locus coeruleus *via* collaterals from the spinothalamic tract (4). Historically, the PLR has been determined subjectively by the examiner. This brings about inaccuracies, not only due to inter-examiner inconsistency, but also a lack of precision of measurement (5). Infrared pupillometry was first introduced in 1989 and used to evaluate the effect of general anesthesia on the human pupillary light reflex (6). Infrared pupillometry involves exposing the eye to infrared light and measuring the reflected image on an infrared sensor. Pupil size and multiple components of the pupillary light reflex and pupillary reflex dilation (PRD) are calculated and subsequently displayed for the user (5)

The primary clinical applications of portable infrared pupillometry include the assessment of brain function during and after trauma, but recently, other uses have been described in humans, such as measuring nociception and pain, particularly intra- and post-operatively. Additional future applications of pupillometry could also include measurement of stress or fear responses, for example. It is especially difficult to assess nociception and pain in children or in patients who are non-verbal, such as those encountered in veterinary medicine. Nociception is the neural process of encoding noxious stimuli, leading to autonomic responses that can potentially be used as measures to quantify nociception and response to analgesic therapy (7). Methods such as heart rate variability, skin conductance, pupillary dilation, and electroencephalography (EEG) have been tested to quantify intraoperative nociception (8). Several studies in humans have documented the value of pupillometry as a guide for analgesia requirement in awake patients (9–11) and in those who are sedated or unconscious (12–14). For example, Aissou et al. (9) showed that shortly after recovery from anesthesia and surgery human patients exhibited pupillary dilation when pressure was applied beside the incision, which correlated with verbal pain ratings before and after morphine titration. In patients under general anesthesia, a unilateral popliteal sciatic nerve block led to a blunted pupillary reflex dilation response to a noxious stimulus, in contrast to the pupillary response elicited by a noxious stimulus to the leg without a nerve block (12).

There is limited evidence for the use of pupillometry in measuring pain in veterinary species or studies reporting the use of an automated infrared pupillometer in animals. One study explored the feasibility of using automated pupillometry in dogs and pupillometry responses of dogs under anesthesia (15). Reference intervals for pupillometry have not been established in dogs. Doing so is an essential first step to facilitate future research exploring pupillometry as a pain assessment method in dogs. The aim of this prospective study is to develop *de novo* pupillometry reference intervals in healthy dogs.

## Methods

The study was reviewed and approved by the Veterinary Sciences Animal Care Committee (University of Calgary, study ID: AC21-0159). Written, informed consent was obtained from owners. Dogs were recruited from 3 sources: the University of Calgary's veterinary teaching colony (Beagles), from the staff of a local referral hospital and from staff and students of the University of Calgary Faculty of Veterinary Medicine. Data were collected between November 2021 and February 2022.

Before examination and testing, dogs were habituated to the area by being allowed to roam freely and explore (~5 min). Each recruited dog underwent an ophthalmic examination performed by a board-certified veterinary ophthalmologist (KCR), consisting of slit lamp biomicroscopy (Keeler PSL Classic Portable Slit Lamp, Malvern, PA, USA), indirect ophthalmoscopy (Volk Pan Retinal 2.2 Classic Lens, Mentor, OH, USA; Keeler Vantage Plus Convertible Slimline Wireless Binocular Indirect Ophthalmoscope, Malvern, PA, USA), Schirmer tear test, fluorescein test and measurement of intra-ocular pressure (iCare Tonovet Plus, Vantaa, Finland). Inclusion criteria were animals with normal ophthalmic exams and no indication of pain or sedation. Dogs with lenticular sclerosis were included in the study, as this is a normal age-related change. Exclusion criteria were previous or current ocular abnormalities including iris atrophy, persistent pupillary membranes, uveitis, glaucoma, cataracts, and keratoconjunctivitis sicca. Additional exclusion criteria were systemic illness, ophthalmic or oral medications, history of chronic pain, evidence of acute pain at time of data collection, and increased stress or behavioral difficulties (defined as resisting restraint) at the time of data collection. In addition, a physical exam, including body condition scoring (16), was performed for each dog before the study. Pain was assessed using the short-form of the Glasgow Composite Measure Scale, which is a behavior-based composite measure scale for assessing acute pain in dogs (17). This scale includes assessment of behavior when undisturbed and during observer interaction. The level of sedation was determined using a validated sedation scale that comprises the following items: spontaneous posture, palpebral reflex, eye position, jaw and tongue relaxation, response to noise (handclap), resistance when laid in lateral recumbency, and general appearance/attitude (18). Pain and level of sedation were assessed to rule out the presence of pain or sedation, both of which may have affected the pupillometry measurements.

The PLR was measured with a handheld pupillometer (NeurOptics Inc.™ PLR-200 Pupillometer, Irvine, CA, USA), with a measurement accuracy of ± 0.1 mm. During the ophthalmic exam and PLR measurement, minimal restraint was used, with no sedatives or anxiolytics administered. Measurements were obtained between 9 a.m. to 1 p.m. The pupillometer was placed in front of the eye, and measurement began following a flash of white light of fixed intensity (180 uW) and duration (154 ms) to stimulate the PLR. Parameters recorded included maximum pupil diameter before constriction (MAX; mm), minimum pupil diameter at the peak of constriction (MIN; mm), percent constriction [CON; (MAX-MIN)/MAX × 100], latency to onset of constriction (LAT; seconds), average constriction velocity (ACV; mm/s), maximum constriction velocity (MCV; mm/s), average dilation velocity (ADV; mm/s) and time to recover 75% of the initial resting pupil size after attaining peak constriction (T75; seconds). Measurement of the PLR was performed once for each eye. Measurements were obtained in rooms with the lights on. Light levels were measured with a light meter (Dr. Meter™ Model LX1330, Hong Kong ThousandShores Limited, Central HK, China) before obtaining measurements.

All data were analyzed with statistical software (GraphPad Prism version 9.0.0 for Mac OS, GraphPad Software, San Diego, California USA, and R version 4.1.2 (https://www.R-project.org/) with “referenceIntervals” package version 1.2.0 (Finnegan D, https://CRAN.R-project.org/package=referenceIntervals). The ASVCP 2011 statistical guidelines for developing *de novo* reference ranges in veterinary species were adhered to for this study (19). Target sample size (> 40 study subjects) was based on these guidelines. Summary statistics for each of the pupillometry parameters were obtained including the mean, standard deviation, median, minimum and maximum values. A Horn's test for outliers was performed for each parameter. Normality was determined by Shapiro-Wilk test, with a *p*-value of <0.05 considered as statistically significant. Ninety-five percent reference limits were developed using a robust method for all parameters except for T75, which used a parametric method with 90% reference intervals due to the sample size being < 40. Bootstrapping method was used to calculate confidence intervals with 5,000 samples for all parameters. Data analysis was performed by a statistician (GPSK).

Data supporting the results are available in a repository: Pang, Daniel, 2022, “Pupillometry repository data,” https://doi.org/10.7910/DVN/6U1ULB, Harvard Dataverse, V1.

## Results

Seventy-eight healthy dogs were initially recruited for this study, consisting of 39 of spayed females and 39 castrated males. Ten dogs had both eyes excluded due to ocular abnormalities (5 subjects had bilateral cataracts, 4 subjects had bilateral iris atrophy, and 1 subject had bilateral pigmentary uveitis). Three dogs had one eye excluded due to ocular abnormalities (unilateral cataracts). Three dogs had one eye excluded due to the pupil being too dilated for the pupillometer to measure. Two dogs were excluded due to behavior. Age of the included subjects ranged from 6 months to 12 years (median 4.5 years, mean ± SD 4.6 ± 3.0 years). Body mass ranged from 3.2 to 40.9 kg (median 12.7 kg, mean ± SD 16.8 ± 10.0 kg). A variety of breeds were included: Standard Poodle (*n* = 1), Labrador Retriever (*n* = 2), Husky (*n* = 1), Border Collie (*n* = 3), Cocker Spaniel (*n* = 1), French Bulldog (*n* = 1), Rough Collie (*n* = 1), Staffordshire Terrier (*n* = 1), Chihuahua (*n* = 2), Toy Poodle (*n* = 2), Australian Shepherd (*n* = 4), Springer Spaniel (*n* = 1), Doberman (*n* = 1), Boxer (*n* = 1), Stabyhoun (*n* = 2), Norwich Terrier (*n* = 1), German Shepherd (*n* = 2), Bernese Mountain Dog (*n* = 1), Yorkshire Terrier (*n* = 1), Pitbull Terrier (*n* = 1), Golden Retriever (*n* = 1), Beagle (*n* = 21), and Mixed Breed (*n* = 14). All dogs had a pain score of zero (scale range 0–20). One dog had a sedation score of 2 as a result of lying in lateral recumbency during the exam (scale range 0–21). Data from this dog was included (18). All other sedation scores were zero. The light measurements ranged from 209 to 236 Lux. After assessing the data distributions between measurements from both eyes and their mean values, there were no significant differences in the distributions and therefore mean values were used for the purpose of this analysis. A total of 126 eyes (*n* = 61 left, *n* = 65 right) were included for analysis.

The summary statistics, reference limits, and confidence intervals for average MAX (number of dogs (*n* = 66), MIN (*n* = 66), CON (*n* = 66), MCV (*n* = 66), ACV (*n* = 66), ADV (*n* = 56), and T75 (*n* = 36) are reported in Tables 1, 2. ADV and T75 had a reduced sample size as the pupillometer was unable to obtain these measurements for all study subjects. Latency was determined to have 3 suspected outliers and ADV was determined to have 5 suspected outliers as per the Horn's test for outliers; however, these data were included in the reference intervals as there were no ocular abnormalities or behavioral changes reported during data collection. All measurements were considered Gaussian in distribution except for MAX, LAT, and ADV.

**Table 1:** Summary statistics for pupillometry measurements.

	**Summary statistics**					
**Variable**	***n* (eyes)**	**Mean**	**Standard deviation**	**Median**	**Minimum**	**Maximum**
Max (mm)	126	8.53	1.30	8.65	4.80	10.00
Min (mm)	126	6.57	1.41	6.65	3.30	9.40
Con (%)	126	−23.51	6.99	−23.00	−36.50	−6.00
Lat (seconds)	126	0.22	0.05	0.21	0.12	0.39
ACV (mm/s)	126	−4.46	0.93	−4.49	−6.81	−2.10
MCV (mm/s)	126	−6.07	1.17	−5.98	−8.66	−2.75
ADV (mm/s)	82	0.88	0.48	0.79	0.02	2.30
T75 (seconds)	44	1.93	0.76	1.72	0.68	3.92

**Table 2:** Statistics, reference intervals and confidence intervals for pupillometry measurements.

**Parameter**	**Horn's test for outliers**	**Shapiro-Wilk normality test**	***p*-value**	95% RI*		**90% CI for Lower RI**		**90% CI for Upper RI**	
MAX (mm)	None	Fail	0.0002	6.05	11.30	5.51	6.62	10.82	11.73
MIN (mm/s)	None	Pass	0.7140	3.76	9.44	3.22	4.23	9.01	9.90
CON (%)	None	Pass	0.2573	−37.89	−9.64	−40.58	−35.87	−12.10	−7.27
LAT (seconds)	Yes	Fail	0.0001	0.11	0.30	0.09	0.13	0.28	0.33
ACV (mm/s)	None	Pass	0.4977	−6.39	−2.63	−6.71	−6.05	−2.99	−2.26
MCV (mm/s)	None	Pass	0.5780	−8.45	−3.75	−8.87	−8.05	−4.15	−3.29
ADV (mm/s)	Yes	Fail	0.0021	−0.21	1.77	−0.44	−0.02	1.54	2.02
T75 (seconds)	None	Pass	0.1086	0.49	3.20	0.16	0.78	2.85	3.67

^*^95% RI (Reference Interval) was calculated, except for T75 due to sample size < 40, in which case 90% RI was applied. CI, confidence interval.

## Discussion

The reference intervals successfully determined in the current study are a necessary first step in establishing the role of pupillometry in dogs. Pupillometry reference intervals have been established for pediatric and adult humans. In a study of 155 healthy individuals between 6 and 64 years of age reported the following: MAX 5.8 ± 0.8 mm (mean ± SD) (range 4.1–7.7), CON 55.8 ± 7.1% (50.2–60.5), LAT 263.5 ± 38.6 ms (126–319), ACV 5.8 ± 0.9 mm/s (4.1–7.9), and ADV 2.1 ± 0.5 mm/s (1.36–3.84) (20). Comparing these human data to those reported in the current study, the canine MAX, ACV, and ADV were greater, whereas CON and LAT were decreased. These changes may reflect differences in PLR physiology and highlight the importance of developing species-specific reference intervals for various pupillometry values. To the authors' knowledge, there has not been a study directly comparing iris innervation between humans and canines, however, comparisons of adrenergic innervation of the iris and ciliary body across other species (mammalian, reptile, avian) has been reported, and identified species specific differences among the adrenergic receptors present in these tissues (21). Tekin et al. (20) found that static and dynamic pupillometry measurements did not vary significantly between males and females; however, age was inversely and moderately correlated with each of the static pupillometric measurements. MAX (*p* < 0.001, *r* = −0.63), ACV (*p* < 0.001, *r* = −0.35), and ADV (*p* < 0.001, *r* = −0.34) values were inversely and moderately correlated with age, and LAT (*p* = 0.002, *r* = 0.29) was positively and moderately correlated with age. Partitioning into subclass based on physiological parameters should only be performed when there are > 40 individuals per subclass, and therefore could not be achieved in this study (19).

In humans, based on an established correlation between pain and changes in pupillometry variables, pupillometry has the potential to quantify postoperative pain and guide analgesic therapy. Pupillometry is attractive as a means of pain assessment as it is non-invasive, rapid to perform and does not require a verbal response. However, more research is needed to establish the role of pupillometry in pain management, with a limited number of existing high quality clinical studies (22–24). As previously discussed, the pupil is controlled by the autonomic nervous system, with two iris muscles, which oppose each other: the dilator muscle which is sympathetically innervated, and the iris sphincter muscle, which is parasympathetically innervated. The sympathetic neuromuscular synapse in the iris is an α_1_-adrenergic junction, and therefore, catecholamines such as epininephrine and norepinephrine produce pupillary dilation (5). The pupillary sphincter, however, contains cholinergic synapses between the muscle and the Edinger-Westphal nucleus, which is one of two nuclei of the oculomotor nerve (5). The impact of stress or pain on autonomic tone were mitigated in this study by excluding the presence of pain and excluding dogs that were stressed. The presence of physiological stress should be considered when obtaining pupillometry measurements, as stress-induced pupillary responses could be misinterpreted as pain.

In veterinary medicine, there are very few pupillometry studies, and none to our knowledge that assess the use of pupillometry as a measure of pain in dogs or have established pupillometry reference intervals. To the authors' knowledge, the first pupillometry study was performed in 105 conscious beagle dogs in the 1970s (25). That study was performed using a fundus camera with an attached eyepiece graticule. Pupil diameters were measured under two different light intensities (150–200 lux and 100–1400 lux) and time-effect curves were plotted after the Beagles received either atropine or propantheline. This study determined a mydriatic pupil diameter of 11.02 ± 0.83 mm in males and 10.69 ± 0.82 mm in females, and a miotic pupil diameter of 1.02 ± 0.32 mm in males and 1.01 ± 0.41 mm in females. The mydriatic pupil diameter falls within our determined reference intervals. The miotic pupil diameter falls below our reference interval. The author, while stating that it seemed to be a promising technique to measure pupil diameter, did note however that a limitation of this technique was that it was difficult to ensure observations were carried out under evenly diffused illumination. A more recent study reported quantitative assessment of the PLR in dachshunds using a custom apparatus and recording system. The average baseline pupil diameter measured in conscious, unrestrained dogs in this study was 10.06 ± 0.54 mm (26) which also falls within the reference interval of the current study. In 2014, a study was performed using an infrared pupillometer similar to the one used in the current study. Some parameters, including CON, ACV and MCV decreased significantly (*p* < 0.05) in anesthetized dogs vs. non-anesthetized dogs (15). The mean values of non-anesthetized dogs reported in this published study, including MAX (9.60 ± 0.57 mm), MIN (7.06 ± 0.91 mm), CON (26.6 ± 6.57%), LAT (0.28 ± 0.07 s), ACV (3.59 ± 0.85 mm/s), MCV (5.83 ± 1.40 mm/s), and ADV (1.63 ± 0.55 mm/s) are again within the reference intervals determined in this study.

The following limitations of the current study should be considered. One is that the pupillometer used is unable to measure pupils with a diameter exceeding 10 mm. As a result, some of our established reference intervals such, as MAX, are skewed by this, as there were three dogs that had one eye excluded due to the device being unable to obtain accurate measurements for the mydriatic pupil. The pupillometer used in this study was developed for humans. As mentioned above, a previous study in humans had shown that the MAX was 5.8 ± 0.8 mm (mean ± SD) and the upper range did not exceed 7.7 mm (20), whereas in this study the canine MAX was determined to be 8.53 ± 1.3 mm (mean ± SD) with three eyes identified with mydriatic pupils > 10 mm. Therefore, the true reference interval for maximum pupil size is likely greater than that reported in this study. There is evidence of significant interspecies variation with regards to the neurophysiology of the PLR. For example, the cat iris appears to be highly responsive to injected epinephrine, whereas the primate iris fails to respond (27). Ciliary innervation varies between species. In the dog, there are 5–8 ciliary nerves, which contain mixed parasympathetic, sympathetic, and sensory afferent fibers (28). In cats, there are two short ciliary nerves that contain only parasympathetic fibers (29). These physiologic differences may account for the mydriatic pupils that were unable to be measured by the pupillometer, and this should be taken into account if this pupillometer is used in additional canine studies. Future studies should consider interspecies variation and effects of sedative, anesthetic, and analgesic agents on the PLR. Previous studies have reported that in dogs, the administration of intravenous medetomidine (30), morphine (31) and fentanyl (32) decreases pupil size. However, in the cat, opioids such as morphine (33) and sufentanil (34) appear to produce mydriasis. In this study, dogs were not excluded if they had an iris color other than brown. Although the effect of iris color on the pupillary light reflex in dogs has not been evaluated, one study in humans showed that there was an increase on constriction amplitude, re-dilation velocity and constriction velocity in brown irides compared to blue irides (35). These variations should be considered as future pupillometry studies in animals are pursued.

In conclusion, this study has determined reference intervals for automated pupillometry parameters in healthy dogs. Future studies using automated pupillometry should consider the potential for limited capability to measure mydriatic canine pupils and the risk of factors affecting autonomic tone (such as stress) interfering with measurements. These reference intervals form the basis of future work to establish the role of pupillometry in nociception and pain assessment in dogs.

## Data availability statement

The datasets presented in this study can be found in online repositories. The names of the repository/repositories and accession number(s) can be found at: https://doi.org/10.7910/DVN/6U1ULB.

## Ethics statement

The animal study was reviewed and approved by Veterinary Sciences Animal Care Committee (University of Calgary, Study ID: AC21-0159). Written informed consent was obtained from the owners for the participation of their animals in this study.

## Author contributions

EM was responsible for data acquisition and manuscript drafting and revisions. KC-R was responsible for data acquisition and manuscript editing. GK was responsible for statistical analysis and manuscript editing. DP was responsible for the conception of the study and manuscript drafting and editing. All authors contributed to the article and approved the submitted version.

## Funding

This study was funded by the University of Calgary Faculty of Veterinary Medicine Intern Research Fund.

## Conflict of interest

The authors declare that the research was conducted in the absence of any commercial or financial relationships that could be construed as a potential conflict of interest.

## Publisher's note

All claims expressed in this article are solely those of the authors and do not necessarily represent those of their affiliated organizations, or those of the publisher, the editors and the reviewers. Any product that may be evaluated in this article, or claim that may be made by its manufacturer, is not guaranteed or endorsed by the publisher.
